# Beneficial Effect of Heat-Killed *Lactiplantibacillus plantarum* L-137 on Skin Functions in Healthy Participants: A Randomized, Placebo-Controlled, Double-Blind Study

**DOI:** 10.3389/fmed.2022.912280

**Published:** 2022-07-06

**Authors:** Rieko Yoshitake, Hiroko Nakai, Manato Ebina, Kengo Kawasaki, Shinji Murosaki, Yoshitaka Hirose

**Affiliations:** Research and Development Institute, House Wellness Foods Corp., Itami, Japan

**Keywords:** probiotics, immune function, gut-skin axis, water content, skin barrier, quality of life

## Abstract

To determine whether consuming heat-killed *Lactiplantibacillus plantarum* L-137 (HK L-137) influences skin functions, we performed a randomized, placebo-controlled, double-blind study in healthy participants who were conscious of dry skin. A total of 80 healthy participants (20 men, 60 women; mean age, 47.3 years) were assigned to receive a tablet containing HK L-137 or a placebo tablet daily for 12 weeks. Every 4 weeks, the skin water content and transepidermal water loss (TEWL) were measured at the forearm and face, and participants completed two skin-related questionnaires, the Dermatology Life Quality Index and a self-evaluation. The HK L-137 group tended to show greater increases from baseline of water content at the forearm and larger decreases of TEWL at the face. The total scores of both questionnaires improved significantly more in the HK L-137 group. Water content and TEWL improved significantly in participants in the HK L-137 group who were above the median age of study participants or had relatively dry skin. These findings suggest that daily HK L-137 intake can improve dry skin, thereby contributing to skin satisfaction.

## Introduction

The skin provides a life-sustaining interface between the body and the environment and has a strong barrier function that plays important roles in not only protecting the skin from various external factors, such as pathogens, ultraviolet radiation, and allergens, but also preventing water loss. Defects in the barrier function cause an abnormal skin condition known as dry skin, which can have a rough, scaly, and flaky surface and is often accompanied by sensations of itching, burning, stinging, and a feeling of tightness. These symptoms can cause not only physical but also psychological trauma and decrease the quality of life (QOL). The causes of skin barrier dysfunction are still not fully understood, but major contributors include decreased moisturizing ingredients, such as natural moisturizing factors, ceramides, and hyaluronan (HA), and disordered structures in the stratum corneum (SC) and the granular layer (1, 2). Under these abnormal conditions, irritants, allergens, and pathogens can enter the skin and cause skin inflammation; such inflammation, especially type 2 responses, may be critical for the skin barrier defect (3).

T helper (Th) 2 cytokines are known to impair formation of the skin barrier by inhibiting keratinocyte differentiation and diminishing tight junctions (4–6). Stefan et al. demonstrated that Th2-derived cytokines disturbed the cornified envelope and tight junction proteins in filaggrin-deficient skin equivalents (7). These results suggest that Th2 inflammation may exacerbate the impaired skin barrier to form a vicious cycle, thereby accelerating dry skin. On the other hand, other studies showed that Th1 cytokines upregulated HA, ceramide, and filaggrin in cultured normal human keratinocytes (8–10). Thus, blocking excessive Th2-type immune responses might be a feasible approach to improve the barrier function of dry skin.

Many studies have demonstrated the effects of probiotics on regulating immune function and improving various diseases, including infection, cancer, gastrointestinal disorders, and metabolic syndrome (11). Probiotics also influence skin function and protect against atopic dermatitis, psoriasis, acne, and skin aging (12). In hairless mice, oral administration of *Lactiplantibacillus plantarum* HY7714 inhibited UVB-induced transepidermal water loss (TEWL) by increasing de novo synthesis of ceramides (13). Another study suggested that *L. plantarum* K8, which can suppress the Th2 response, attenuated the decreased barrier function in mice with atopic dermatitis (14). Clinical studies also showed protective effects of dietary supplements containing heat-killed *Lacticaseibacillus paracasei* K71 in adult patients with atopic dermatitis (15) and of dietary supplements containing *Bifidobacterium breve* in healthy adult women (16).

*Lactiplantibacillus plantarum* L-137(former *Lactobacillus plantarum* L-137) was originally isolated from a fermented Southeast Asian dish made from fish and rice (17). In a mouse model of food allergy, the administration of the heat-killed *L. plantarum* strain L-137 (HK L-137) suppressed immunoglobulin E production against a natural antigen (18), and in mice transplanted with syngeneic tumor cells, it inhibited tumor growth, which correlated with its ability to produce interleukin (IL)-12 (19). Clinical studies showed that daily intake of HK L-137 improved health-related QOL in healthy participants (20) and reduced the incidence of upper respiratory tract infection in participants with high levels of stress (21). Moreover, the aforementioned studies found that HK L-137 enhanced Th1-related immune functions in healthy participants, as evaluated by increased concanavalin A-induced proliferation and percentages of interferon (IFN)-γ and IL-4-producing CD4 T cells (Th1:Th2 ratio) among peripheral blood mononuclear cells (20). These results suggest that HK L-137 is a potent inducer of IL-12 that promotes activation of Th1-related immune responses. HK L-137 was also reported to increase HA production by inducing IFN-γ and tumor necrosis factor (TNF)-α in fibroblasts and to suppress a loss of skin moisture content in mice (22).

## Materials and Methods

### Participants

Healthy persons aged 35–54 years who were conscious of dry skin were recruited in November 2020, and their eligibility for the study was assessed. Exclusion criteria included the following: (1) consumption of foods, supplements, and medicines rich in lactic acid bacteria at least 3 times a week; (2) having a skin condition that tends to be influenced by wearing a mask (inflammation, dryness, etc.), and impossibility of wearing the mask being used during the study; (3) daily consumption of foods, supplements, and medicines that could affect the condition of the skin; (4) prior cosmetic medical treatments at measurement sites (e.g., injection of Botox, hyaluronic acid, or collagen, and intense pulsed light [photofacial] therapy); (5) prior cosmetic medical treatments at sites other than those to be examined, or hormone replacement therapy within a year before the screening test; (6) prior aesthetic, scrubbing, or hair removal at measurement sites within a month before the screening test or intention to receive such treatment during the study period; (7) history of excessive sunburn, e.g., due to outdoor work, leisure, or exercise, within a month before the screening test or high risk of experiencing excessive sunburn during the study period; (8) daily washing of the measurement sites with materials that act as a strong stimulus to the skin, e.g., nylon towels; (9) routine use of bathwater additives; (10) wounds or inflammatory diseases at the measurement sites that could affect the study results; (11) allergic diseases, such as hay fever or atopic dermatitis; (12) extreme skin condition changes at the measurement sites due to menstruation; (13) unstable work schedule or working night shifts; (14) planning to travel abroad during the study; (15) having asthma or a high risk of having an asthma attack during the study; (16) current or previous history of diabetes; hepatic, kidney, cardiac, gastrointestinal, or vascular disease; or other diseases; (17) possibility of allergic reaction to the test food; (18) current or previous history of diseases that could affect the study results or require medication; (19) screening test values considerably outside the reference ranges; (20) participation in other clinical studies within a month before the study or plan to participate in other clinical studies during this study; (21) pregnancy or breastfeeding or intention to become pregnant; (22) being judged unsuitable for this study based on lifestyle questionnaires; and (23) being judged unsuitable for the study by the investigator or sub-investigator.

Among 164 potential recruits, 80 persons (20 men and 60 women; mean age, 47.3 years) who had a relatively low water content in the SC and high TEWL of the skin and were eligible to be enrolled in the study were randomly assigned to the HK L-137 or control group. Physicians judged the participants as being healthy on the basis of blood and urine samples and a medical consultation.

The sample size was determined from the results of a previous study on the effects of Curcuma longa extract on the water content in the SC (23). The mean within-group changes in that study showed a normal distribution, with a standard deviation of 35.7. Assuming that the true difference of the mean value between the treated and control groups was 29.5, we needed 32 participants per group to be able to reject the null hypothesis (i.e., the population means of both groups are equal) with a power of 0.9. The probability of a Type I error associated with this test of the null hypothesis was 0.05. We initially recruited 40 volunteers per group to allow for an estimated drop-out rate of 20% over the study period.

During the intervention period, participants were instructed to avoid changing their lifestyle from their preintervention patterns as far as possible; this included diet, alcohol consumption, exercise, sleep, smoking, bathing activity, and skincare products. They were also asked to refrain from exposing themselves to direct sunlight outdoors and receiving cosmetic medical treatments. In addition, they were requested to wear a study-specific mask when needed.

This study was registered with the University Hospital Medical Information Network Clinical Trials Registry (UMIN 000042296). It complied with the Declaration of Helsinki. After receiving a full explanation of the study, all participants provided written informed consent.

### Preparation of HK L-137

The study used a commercial preparation of HK L-137 (House Wellness Foods Corporation, Hyogo, Japan) that contains 20% HK L-137 and 80% dextrin. HK L-137 was prepared as described previously (18).

### Experimental Design

The study had a randomized, double-blind, placebo-controlled, parallel-group design. Stratified randomization by age, sex, and water content of the SC and TEWL of the skin was used to assign participants to either group by sequentially numbered sealed envelopes that each contained one treatment randomly generated by a computer program. After grouping, the participants took one tablet per day containing either 50 mg of the HK L-137 preparation (10 mg of HK L-137 and 40 mg of dextrin) or a placebo tablet containing 50 mg of dextrin for 12 weeks. Every 4 weeks, the condition of the skin was evaluated, participants responded to the questionnaires, and other procedures were performed. During the intervention period, participants recorded in diaries both the time at which they ingested the tablet and their subjective symptoms. This study was performed by a contract research organization (EP Mediate, Tokyo, Japan) at Ebisu Skin Research Center from December 2020 to March 2021, which corresponded to the winter season and therefore has particularly low humidity. In addition, blood and urine samples were collected by the Medical Station Clinic (Tokyo, Japan) at screening, and the biochemical and hematology tests and urinalysis were subsequently performed by an external clinical laboratory (LSI Medience, Tokyo, Japan).

### Measurement of Skin Parameters

The water content of the SC was measured as the primary efficacy outcome, and the TEWL was measured as the secondary efficacy outcome. Participants were requested to avoid hair removal at the measurement sites (except shaving in men) for 2 weeks prior to the study examination and to avoid visiting a spa and using bathwater additives for 1 week prior to the examination. They were instructed to go to sleep before midnight and to refrain from drinking alcohol and using any facial masks and body lotion from the day before the examination. Furthermore, they were asked to avoid excessive exercise, bathing, and spicy foods on the day of the examination and to avoid consuming any food or drinks, except water, for 4 h before the examination. Before the examination, participants were requested to wash their face and hands and to remain in a room at a temperature of 21°C with a humidity of 50% for at least 20 min. After acclimation, the water content of the SC of the left cheek and inside left forearm was measured five times with a Skicon-200EX (Yayoi Corporation, Tokyo, Japan); the maximum and minimum values were deleted, and the mean value was used. The TEWL of the skin of the left cheek and inside left forearm was measured eight times with a Tewameter TM300 (Courage + Khazaka Electronic GmbH, Cologne, Germany); the maximum and minimum values were deleted, and mean value was used.

### Questionnaires

The Dermatology Life Quality Index (DLQI) and participants' overall satisfaction with their current skin condition were measured the secondary efficacy outcome. DLQI was used to measure how much a skin problem had affected the participants' QOL over the previous 7 days (24). The index consists of 10 items that are scored from 0 to 3 (0 = “not at all” or “not relevant”; 1 = “a little”; 2 = “a lot”; 3 = “very much”). The sum of the scores for the 10 items produces a DLQI summary score between 0 and 30, with higher scores indicating lower health-related QOL. The sums of the scores for 1 to 2 items each are combined to form subdomains on 6 aspects (symptoms/feelings, daily activities, leisure, work/school, personal relationships, and treatment). In addition, participants' overall satisfaction with their current skin condition was assessed on a Likert scale ranging from 1 to 5 (1 = “very dissatisfied”; 2 = “somewhat dissatisfied”; 3 = “neither satisfied nor dissatisfied”; 4 = “somewhat satisfied”; 5 = “very satisfied”).

### Stratified Analyses

Aging and dryness are a typical cause of skin dysfunction because the skin barrier, turnover, moisture level, and immune homeostasis are disordered in these conditions (25–27). Therefore, the skin condition of older participants or those with dry skin was expected to be worse. To evaluate the effects of HK L-137 in such participants, we performed two stratified analyses: (1) in those above the median age of all study participants (i.e., those aged ≥ 48 years old) and (2) in those with a water content of the forearm SC in the bottom half of the values measured at baseline (≤55.500 μS) and a TEWL of the forearm skin surface in the top half of the values measured at baseline (≥9.705 g/h m^2^).

### Statistical Analysis

IBM SPSS statistics version 25 software was used for statistical analyses. Values are shown as the mean ± SD. The baseline values were compared between the two groups with the Chi-square test or unpaired Student's *t*-test. The means of the measured values of water content and TEWL were analyzed by repeated measure analysis of variance (ANOVA), and mean values of the two groups were compared at each time point with the unpaired Student's *t*-test. Changes from baseline of all outcomes were analyzed by two-way ANOVA, and the mean changes from baseline in the two groups were compared at each time point with the unpaired Student's *t*-test.

## Results

### Baseline Characteristics and Drop-Outs

**Figure 1:**
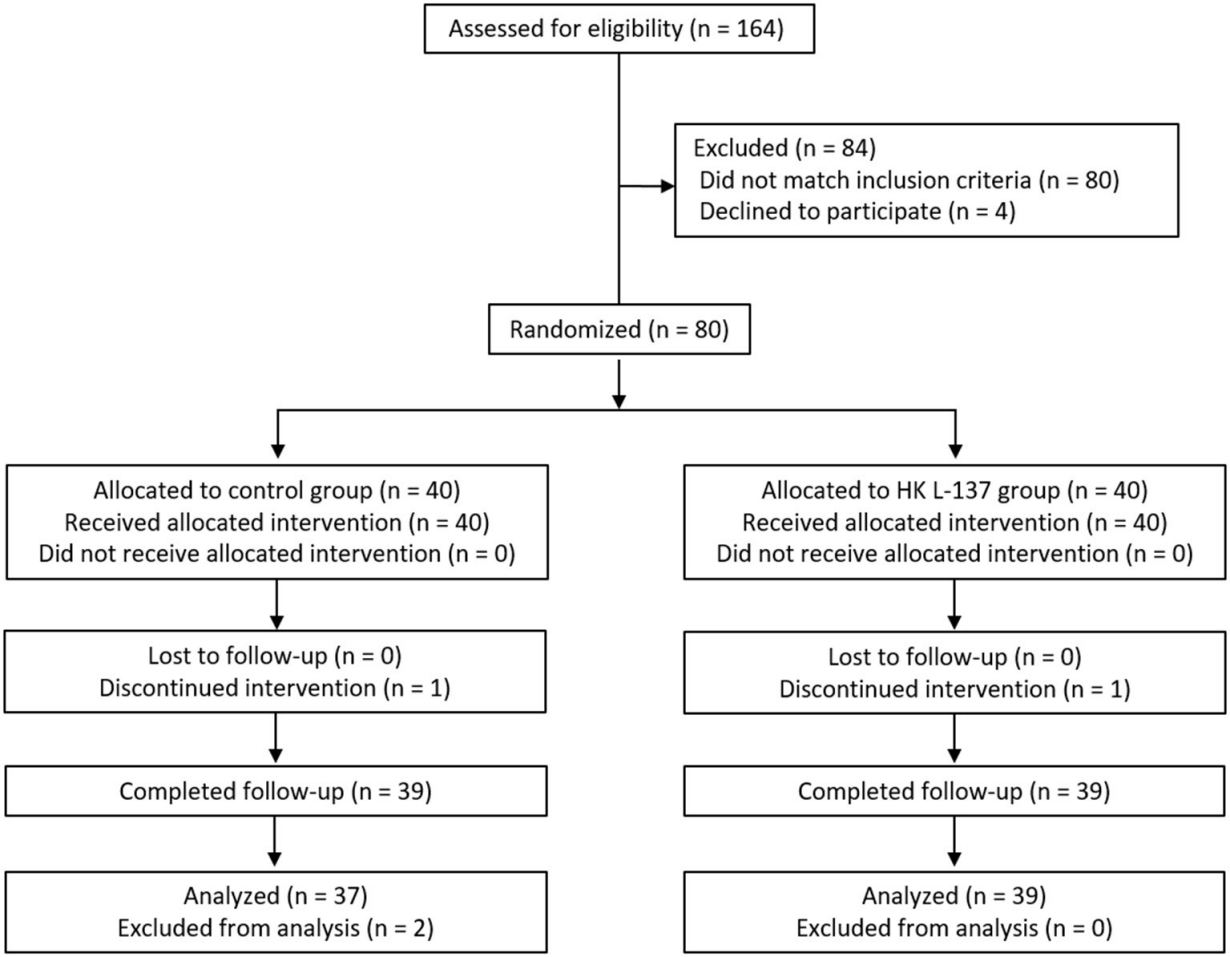
Study flow diagram (CONSORT 2010).

Baseline characteristics showed no significant differences between the HK L-137 and control groups (Table 1). Two participants dropped out before completing the study: one participant in the control group because of personal reasons, and one participant in the HK L-137 group because of cerebral infarction. In addition, two participants in the control group who completed the study were excluded from the analysis because they developed conditions or received treatments that could considerably affect skin function: One developed an allergic disease similar to hay fever, and the other developed tenosynovitis and received a steroidal anti-inflammatory drug by injection. Therefore, a total of 76 participants were included in the statistical analysis (Figure 1).

**Table 1:** Baseline participant characteristics.

	**Control group**	**HK L-137 group**
*N*	37	39
**Sex** ^**a**^		
Male, *n*	10	9
Female, *n*	27	30
Age, year^b^	47.5 ± 5.1	47.1 ± 4.1
BMI^b^	21.0 ± 2.7	20.7 ± 2.5
**Water content** ^**b**^		
Face	99.0 ± 51.5	111 ± 60.8
Forearm	55.3 ± 19.1	60.5 ± 19.7
**TEWL** ^**b**^		
Face	18.9 ± 6.05	18.6 ± 7.17
Forearm	9.75 ± 1.78	9.82 ± 2.03
**DLQI** ^**b**^		
Symptoms/feelings	0.57 ± 0.65	0.41 ± 0.72
Daily activities	0.14 ± 0.42	0.05 ± 0.22
Leisure	0.00 ± 0.00	0.03 ± 0.16
Work/school	0.08 ± 0.28	0.08 ± 0.27
Personal relationships	0.03 ± 0.16	0.03 ± 0.16
Treatment	0.03 ± 0.16	0.03 ± 0.16
Summary score	0.84 ± 1.24	0.62 ± 1.31
Overall satisfaction with the current skin condition^b^	2.43 ± 0.93	2.31 ± 0.83

*BMI, body mass index; DLQI, Dermatology Life Quality Index; TEWL, transepidermal water loss*.

*Unless otherwise indicated, values are the mean ± SD*.

a *Comparison between 2 groups by the Chi-square test*.

b *Comparison between 2 groups by the unpaired Student's t-test*.

### Effects of HK L-137 on the Water Content of the SC and TEWL of the Skin

The results of the measurement of water content and TEWL at weeks 0, 4, 8, and 12 and the changes from baseline are shown in Table 2. The water content of the SC, primary efficacy outcome, increased in both groups during the study because the study was begun at a season with low humidity, but the water content at the forearm tended to be higher in the HK L-137 group (*p* = 0.074), and the increases from baseline in the water content at the forearm also tended to be larger in the HK L-137 group (*p* = 0.096) over the study period. Compared with the control group, the HK L-137 group tended to show a greater decrease from baseline in the TEWL at the face (*p* = 0.050) over the study period. In addition, the water content at the forearm was significantly higher in the HK L-137 group than in the control group at week 4 (*p* = 0.044) and tended to be higher at week 8 (*p* = 0.090).

**Table 2:** Effects of heat-killed *Lactiplantibacillus plantarum* L-137 on the water content of the stratum corneum and transepidermal water loss of the skin.

						**Two-way ANOVA^a^**		
		**Baseline**	**4 weeks**	**8 weeks**	**12 weeks**	**Group**	**Time**	**Interaction**
Water content at the face, μS	Control	99.0 ± 51.5	121 ± 62.1	136 ± 66.0	157 ± 69.9	0.371	0.000	0.985
	HK L-137	111 ± 60.8	135 ± 71.1	150 ± 71.2	170 ± 71.8			
Water content at the forearm, μS	Control	55.3 ± 19.1	58.1 ± 19.6	60.9 ± 21.3	66.2 ± 24.3	0.074	0.000	0.594
	HK L-137	60.5 ± 19.7	68.3 ± 23.6^*^	71.9 ± 32.7^†^	74.1 ± 27.9			
TEWL at the face, g/h m^2^	Control	18.9 ± 6.05	18.1 ± 5.61	18.1 ± 6.48	19.1 ± 5.33	0.403	0.001	0.469
	HK L-137	18.6 ± 7.17	17.0 ± 5.67	16.5 ± 5.85	17.8 ± 5.84			
TEWL at the forearm, g/h m^2^	Control	9.75 ± 1.78	9.06 ± 2.71	8.62 ± 2.17	10.0 ± 2.18	0.996	0.000	0.778
	HK L-137	9.82 ± 2.03	8.90 ± 1.74	8.52 ± 1.76	10.2 ± 1.89			
Δ Water content at the face, μS	Control		22.1 ± 28.6	36.9 ± 33.7	58.5 ± 39.9	0.809	0.000	0.980
	HK L-137		24.2 ± 30.9	38.3 ± 32.7	58.4 ± 43.5			
Δ Water content at the forearm, μS	Control		2.78 ± 9.03	5.58 ± 14.2	10.8 ± 16.6	0.096	0.126	0.893
	HK L-137		7.87 ± 21.5	11.4 ± 28.2	13.6 ± 26.5			
Δ TEWL at the face, g/h m^2^	Control		−0.80 ± 3.59	−0.86 ± 3.20	0.14 ± 3.65	0.050	0.164	0.934
	HK L-137		−1.69 ± 4.12	−2.19 ± 3.91	−0.81 ± 5.34			
Δ TEWL at the forearm, g/h m^2^	Control		−0.69 ± 1.64	−1.13 ± 1.65	0.29 ± 1.55	0.645	0.000	0.784
	HK L-137		−0.91 ± 1.33	−1.29 ± 1.28	0.39 ± 1.77			

*ANOVA, analysis of variance; HK L-137, heat-killed *Lactiplantibacillus plantarum* L-137; TEWL, transepidermal water loss*.

*Values are the mean ± SD*.

* *P < 0.05*,

† *P < 0.10: significant difference of the mean value in the test group vs the control group (unpaired Student's t-test)*.

a *Significant differences were evaluated by 2-way ANOVA (mean values, repeated measure ANOVA; changes from baseline, 2-way ANOVA)*.

### Effects of HK L-137 on Health-Related QOL

The changes from baseline in the DLQI scores and level of satisfaction with the condition of the skin are shown in Table 3. Among the six subscales of the DLQI, the scores for daily activities, leisure, and personal relationships showed a decrease from baseline that was significantly larger or tended to be larger in the HK L-137 group than in the control group over the study period (daily activities, *p* = 0.045; leisure, *p* = 0.008; and personal relationships, *p* = 0.058). In contrast, the change in the scores on the subscales for symptoms/feelings, work/school, and treatment showed no significant differences between the two groups. Compared with the control group, the HK L-137 group showed a significantly greater decrease in the DLQI total score (*p* = 0.038) and a significantly greater increase in the score for overall satisfaction with the current skin condition (*p* = 0.007) over the study period. In addition, the scores for leisure and personal relationships showed a decrease from baseline that was significantly larger or tended to be larger in the HK L-137 group than in the control group at week 8 (leisure, *p* = 0.029; and personal relationships, *p* = 0.099). The total DLQI score tended to be lower in the HK L-137 group than in the control group (*p* = 0.097) and the score for the overall satisfaction with current skin condition tended to be higher (*p* = 0.070) at week 8.

**Table 3:** Effects of heat-killed *Lactiplantibacillus plantarum* L-137 on health-related quality of life.

		**4 weeks**	**8 weeks**	**12 weeks**	**Two-way ANOVA** ^**a**^		
					**Group**	**Time**	**Interaction**
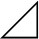 DLQI symptoms/feelings	Control	0.19, 1.20	−0.08, 1.01	−0.22, 0.82	0.612	0.074	0.569
	HK L-137	0.05, 0.65	−0.23, 0.58	−0.10, 0.79			
Daily activities	Control	0.22, 0.71	0.16, 0.60	0.16, 0.65	0.045	0.604	0.959
	HK L-137	0.10, 0.45	0.03, 0.28	0.00, 0.23			
Leisure	Control	0.11, 0.31	0.16, 0.50	0.16, 0.69	0.008	0.975	0.536
	HK L-137	0.05, 0.22	−0.03, 0.16^*^	0.00, 0.00			
Work/school	Control	0.14, 0.42	0.14, 0.35	0.03, 0.37	0.102	0.262	0.897
	HK L-137	0.03, 0.43	0.05, 0.39	−0.03, 0.28			
Personal relationships	Control	−0.03, 0.16	0.08, 0.36	0.03, 0.16	0.058	0.280	0.280
	HK L-137	−0.03, 0.16	−0.03, 0.16^†^	−0.03, 0.16			
Treatment	Control	0.08, 0.28	0.05, 0.23	0.03, 0.16	0.131	0.189	0.884
	HK L-137	0.05, 0.22	0.00, 0.00	0.00, 0.00			
Total score	Control	0.70, 2.21	0.51, 2.52	0.19, 2.28	0.038	0.277	0.806
	HK L-137	0.26, 1.45	−0.21, 0.86^†^	−0.15, 0.96			
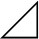 Overall satisfaction with the current skin condition	Control	0.05, 0.97	0.30, 0.91	0.62, 1.16	0.007	0.003	0.961
	HK L-137	0.38, 1.02	0.72, 1.07^†^	0.97, 1.01			

*ANOVA, analysis of variance; DLQI, Dermatology Life Quality Index; HK L-137, heat-killed Lactiplantibacillus plantarum L-137*.

*Values are the mean ± SD*.

* *p < 0.05*,

† *p < 0.10: significant difference of the mean value in the test group vs. the control group (unpaired Student's t-test)*.

a *Significant differences were evaluated by 2-way ANOVA*.

### Analysis Stratified by Age and Skin Parameters

The results of the two stratified analyses (25–27) in participants who were above the median age in this study (i.e., who were ≥48 years old) and who at baseline had a water content of the forearm SC in the bottom half of the measurements (≤ 55.500 μS) and a forearm TEWL in the top half (≥9.705 g/h m^2^) are shown in Table 4.

**Table 4:** Analysis of water content of the stratum corneum and transepidermal water loss of the skin stratified by baseline age and skin parameters.

		**4 weeks**	**8 weeks**	**12 weeks**	**Two-way ANOVA** ^**a**^		
					**Group**	**Time**	**Interaction**
**Age ≥48 years** ^**b**^							
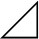 Water content at the face, μS	Control	17.0, 21.9	32.8, 28.1	54.3, 40.4	0.094	0.000	0.999
	HK L-137	27.0, 24.5	42.2, 25	63.9, 41.5			
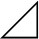 Water content at the forearm, μS	Control	1.91, 9.26	9.53, 13.4	15.5, 19.0	0.013	0.152	0.675
	HK L-137	15.3, 25.7^*^	22.0, 34.4	20.7, 27.8			
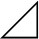 TEWL at the face, g/h m^2^	Control	0.05, 3.98	−0.39, 3.37	0.54, 3.68	0.002	0.510	0.917
	HK L-137	−1.73, 4.00	−2.81, 2.77^*^	−1.84, 4.97^†^			
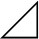 TEWL at the forearm, g/h m^2^	Control	−0.77, 1.63	−1.34, 1.52	0.00, 1.27	0.747	0.000	0.812
	HK L-137	−1.07, 1.35	−1.42, 1.29	0.12, 1.76			
**Water content of the forearm skin ≤ 55.500 μS and TEWL of the forearm skin ≥ 9.705 g/h m** ^**2**^ **^c^**							
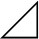 Water content at the face, μS	Control	14.0, 7.38	25.8, 13.3	42.0, 14.5	0.053	0.000	0.407
	HK L-137	15.2, 11.9	40.7, 20.1^†^	64.3, 54.9			
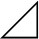 Water content at the forearm, μS	Control	1.21, 8.78	5.21, 11.8	6.61, 12.3	0.002	0.726	0.941
	HK L-137	21.8, 28.1^*^	30.0, 48.3	26.1, 26.6^*^			
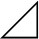 TEWL at the face, g/h m^2^	Control	−0.86, 2.08	−0.20, 3.71	0.76, 3.17	0.029	0.557	0.394
	HK L-137	−1.10, 2.76	−2.78, 1.44	−1.66, 2.19^†^			
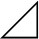 TEWL at the forearm, g/h m^2^	Control	−0.59, 2.37	−1.39, 2.07	0.60, 1.56	0.074	0.037	0.886
	HK L-137	−1.72, 1.53	−1.99, 1.26	−0.59, 2.14			

*ANOVA, analysis of variance; HK L-137, heat-killed Lactiplantibacillus plantarum L-137; TEWL, transepidermal water loss*.

*Values are the mean ± SD*.

* *p < 0.05*,

† *p < 0.10: significant difference of the mean value in the HK L-137 group vs. the control group (unpaired Student's t-test)*.

a *Significant differences were evaluated by 2-way ANOVA*.

b *Control group, n = 22; HK L-137 group, n = 18*.

c *Control group, n = 11; HK L-137 group, n = 7*.

Among the 40 participants aged 48 years or older (mean age, 50.88 years), compared with the control group (*n* = 22), participants in the HK L-137 group (*n* = 18) showed a significantly larger increase in the water content at the forearm (*p* = 0.013), a significantly larger decrease in the TEWL at the face (*p* = 0.002) over the study period, and a tendency for a larger increase in the water content at the face over the study period (*p* = 0.094). In addition, the HK L-137 group showed a significantly larger increase than the control group in the water content at the forearm at week 4 (*p* = 0.028) and a significantly larger decrease in the TEWL at the face at week 8 (*p* = 0.019) and showed a tendency for a larger decrease in the TEWL at the face at week 12 (*p* = 0.089).

Among the 18 participants with a lower water content and higher TEWL at the forearm at baseline (mean age, 47.56 years), over the study period, the HK L-137 group (*n* = 7) showed a significantly larger increase than the control group (*n* = 11) in the water content at the forearm (*p* = 0.002) and a significantly larger decrease in the TEWL at the face (*p* = 0.029) and showed a tendency for a larger increase in the water content at the face and a larger decrease in TEWL at the forearm (*p* = 0.053 and *p* = 0.074, respectively). In addition, compared with the control group, the HK L-137 group showed a significantly larger increase in the water content at the forearm at weeks 4 and 12 (*p* = 0.035 and *p* = 0.049, respectively) and showed a tendency for a larger increase in the water content at the face at week 8 (*p* = 0.076) and a larger decrease in TEWL at the face at week 12 (*p* = 0.098).

### Safety

Side effects and adverse events were assessed in the full analysis set (control group, *n* = 40; HK L-137 group, *n* = 40). In the control group, adverse events occurred in 30 participants: skin symptoms (rough skin, itchy skin, exanthema, or acne) in six participants; gastrointestinal symptoms (stomachache, nausea, diarrhea, or constipation) in six participants; nose or throat symptoms (sore throat, runny nose, stuffy nose, sneeze, or intranasal cyst) in five participants; common cold symptoms in three participants; eye symptoms (itching, dryness, or tiredness) in three participants; headache or nausea in three participants; menstrual pain in two participants; tenosynovitis in one participant; and stomachache, headache, and nausea in one participant. In the HK L-137 group, adverse events occurred in 17 participants: gastrointestinal symptoms (stomachache or diarrhea) in five participants; headache or lower back pain in four participants; menstrual pain in three participants; common cold symptoms in two participants; cerebral infarction in one participant; sore throat in one participant; and fatigue in one participant. An experienced physician rated these adverse events as unrelated to the intervention.

## Discussion

In the present randomized, placebo-controlled, double-blind study, we investigated the effects of HK L-137 on skin moisture and skin barrier function in healthy participants who were conscious of dry skin. The oral intake of HK L-137 increased the water content at the forearm and decreased the TEWL at the face with statistical tendency. In addition, the change from baseline in the total DLQI score and in the score for the overall satisfaction with the current skin condition improved significantly in the HK L-137 group compared with the control group over the study period. In the stratified analysis of participants above the median age, and of participants with a relatively dry forearm (bottom half of the baseline measurement of the forearm water content and top half of the baseline measurement of the forearm TEWL at baseline), the increase of the water content at the forearm and the decrease of TEWL at the face were significantly greater in the HK L-137 group. These results suggest that administration of HK L-137 could augment the skin barrier and improve dry skin in healthy adults, especially in older people or people with dry skin, and thereby improve their satisfaction with their current skin condition and health-related QOL, although the clinical relevance is needed to clarify because of the low DLQI scores at baseline.

HA, a nonsulfated glycosaminoglycan composed of repeating disaccharide units of *N*-acetylglucosamine and glucuronic acid, maintains skin hydration through its capacity to bind and retain water molecules. The major location of HA in the skin is the dermis, but HA has also been found in the epidermis and even in the SC (28). Previous studies showed that HA is closely involved in keratinocyte proliferation and differentiation (29–31) and may participate in epidermal structure and turnover. In addition, several clinical studies reported that ingested HA increases skin moisture and improves treatment outcomes in patients with dry skin (32–34). These reports suggest that augmentation of HA synthesis could induce normalization of epidermal structure and improve skin moisture in people with dry skin. HK L-137 has been shown to enhance skin moisture in the HR-1 hairless mouse and to upregulate mRNA expression of IFN-γ and TNF-α and HA production from primary epidermal cell cultures *in vitro* (22), suggesting that HK L-137 might augment HA synthesis in this study.

Many lactic acid bacteria have been reported to confer skin health benefits, mainly by manipulation of the intestinal microbiota (35). The mechanisms by which improvement of the intestinal microbiome positively affects skin homeostasis are not fully understood, but several reports have demonstrated the existence of communications in the gut-skin axis *via* bacterial metabolites (36), immune system (37), and intestinal barrier (38). HK L-137, a heat-treated dead bacteria has been shown to regulate intestinal microbiota and increase short-chain fatty acids in human subjects (39). Moreover, other studies reported that HK L-137 inhibits plasma lipopolysaccharide-binding protein levels, a marker of intestinal permeability, in diet-induced obese mice (40) and induces intestinal cell growth by activating intestinal function in broiler chickens (41, 42). These suggest HK L-137 might improve intestinal barrier in a direct or indirect manner. A defective intestinal barrier has been reported to cause inflammation in non-intestinal organs, such as liver, fat tissues, and kidney (43), and, in fact, DNA of bacterial intestinal origin can be found circulating in the blood of patients with psoriasis (44). Moreover, phenol and para-cresol, which are produced by intestinal bacteria from the amino acids tryptophan and tyrosine, respectively (45), impair epidermal barrier integrity by reducing the expression of keratin 10 in keratinocytes (46). Accumulating evidence suggests that luminal noxious molecules can permeate into the circulation by disrupting the intestinal tight junction barrier and reach the skin, undermining of skin integrity. Thus, it is possible that HK L-137 might also have positive effects on the epidermal barrier by strengthening the intestinal barrier.

We assessed whether HK L-137 influences psychological conditions because dry skin can have negative consequences on health-related QOL by causing itching, discomfort, and embarrassment about appearance. In the present study, the DLQI score improved significantly in the HK L-137 group despite the low scores at baseline. The DLQI is a health-related QOL scale that focuses on skin-specific QOL measures and is designed for use in dermatology patients. A French large-scale opinion poll demonstrated that the DLQI score was correlated with the severity of sensitive skin in that 80% of persons with sensitive skin declared that they had dry skin (47). Moreover, this study and another studies (48) also showed improvement of the DLQI score in parallel with the increase of the water content, and the satisfaction with the condition of the skin, which suggest that the DLQI could be an applicable questionnaire to investigate the effects of dry skin on health-related QOL even if in healthy subjects. Thus, improvement of dry skin by HK L-137 might increase people's satisfaction with the condition of their skin and health-related QOL.

Aging and dryness are major factors that disturb skin functions such as the skin barrier, turnover, moisturizing, and immune system (25–27). Aging can affect HA amount (49) and gut permeability (50), which is related to skin health, which is why we performed stratified analyses to assess the effects of HK L-137 in older participants or those with dry skin. We evaluated dry skin condition in reference to water content and TEWL at the forearm because this location is less affected by the environment than the face. The medians of the forearm measurements in this study were comparable to those of previous studies (51–53). We found that oral intake of HK L-137 significantly improved the water content at the forearm and the TEWL at the face, especially in older participants and those with dry skin. Previous studies have reported that the cheek has fewer layers of SC than the forearm (54), and shows lower water content and higher TEWL than other sites (55), suggesting that the skin barrier function at the cheek is lower than at other sites. These might lead to different effectiveness of HK L-137 in distinct site. In addition, this study was performed from winter to spring, in which the water content of the SC increased with higher temperatures and with higher relative humidity, and which is why it became difficult to show the effectiveness of HK L-137 on skin functions at week 12.

In conclusion, we demonstrated in healthy volunteers that daily intake of HK L-137 enhances skin moisture at the forearm and tends to upregulate the skin barrier function at the face, as assessed by measuring TEWL. These effects of HK L-137 were also seen in people whose skin condition was expected to be relatively worse, i.e., those above the median age and those with relatively dry skin. In addition, intake of HK L-137 improved satisfaction with the condition of the skin and health-related QOL. Our results suggest that HK L-137 may be useful for preventing and treating dry skin-related disorders.

## Data Availability Statement

The raw data supporting the conclusions of this article will be made available by the authors, without undue reservation.

## Ethics Statement

The studies involving human participants were reviewed and approved by the University Hospital Medical Information Network Clinical Trials Registry. The patients/participants provided their written informed consent to participate in this study.

## Author Contributions

ME, HN, and YH designed this research study. ME and HN conducted the research. RY, KK, YH, and SM analyzed the data. RY wrote the paper. KK, YH, and SM participated in interpretation of the results. All authors read and approved the final manuscript.

## Funding

This study was conducted by a contract research organization (EP Mediate Co., Ltd., Tokyo, Japan) with financial support from House Wellness Foods Corp.

## Conflict of Interest

RY, HN, ME, KK, SM, and YH are employed by House Wellness Foods Corp. This study received funding from House Wellness Foods Corp. The funder had the following involvement with the study, the study design, collection, analysis, interpretation of data, the writing of this article, and the decision to submit it for publication.

## Publisher's Note

All claims expressed in this article are solely those of the authors and do not necessarily represent those of their affiliated organizations, or those of the publisher, the editors and the reviewers. Any product that may be evaluated in this article, or claim that may be made by its manufacturer, is not guaranteed or endorsed by the publisher.
