# Corrigendum: Beneficial effect of heat-killed *Lactiplantibacillus plantarum* L-137 on skin functions in healthy participants: A randomized, placebo-controlled, double-blind study

**DOI:** 10.3389/fmed.2022.1048906

**Published:** 2022-10-18

**Authors:** Rieko Yoshitake, Hiroko Nakai, Manato Ebina, Kengo Kawasaki, Shinji Murosaki, Yoshitaka Hirose

**Affiliations:** Research and Development Institute, House Wellness Foods Corp., Itami, Japan

**Keywords:** probiotics, immune function, gut-skin axis, water content, skin barrier, quality of life

In the published article, there was an error in the legend and footnote for Table 2 as published. The strain's name of lactic acid bacteria was described as “*Lactiplantibacillus* L-137.” The correct name is “*Lactiplantibacillus plantarum* L-137.” The correct legend and footnote appears below.

**Table 2:** Effects of heat-killed *Lactiplantibacillus plantarum* L-137 on the water content of the stratum corneum and transepidermal water loss of the skin.

						**Two-way ANOVA^a^**		
		**Baseline**	**4 weeks**	**8 weeks**	**12 weeks**	**Group**	**Time**	**Interaction**
Water content at the face, μS	Control	99.0 ± 51.5	121 ± 62.1	136 ± 66.0	157 ± 69.9	0.371	0.000	0.985
	HK L-137	111 ± 60.8	135 ± 71.1	150 ± 71.2	170 ± 71.8			
Water content at the forearm, μS	Control	55.3 ± 19.1	58.1 ± 19.6	60.9 ± 21.3	66.2 ± 24.3	0.074	0.000	0.594
	HK L-137	60.5 ± 19.7	68.3 ± 23.6^*^	71.9 ± 32.7^†^	74.1 ± 27.9			
TEWL at the face, g/h m^2^	Control	18.9 ± 6.05	18.1 ± 5.61	18.1 ± 6.48	19.1 ± 5.33	0.403	0.001	0.469
	HK L-137	18.6 ± 7.17	17.0 ± 5.67	16.5 ± 5.85	17.8 ± 5.84			
TEWL at the forearm, g/h m^2^	Control	9.75 ± 1.78	9.06 ± 2.71	8.62 ± 2.17	10.0 ± 2.18	0.996	0.000	0.778
	HK L-137	9.82 ± 2.03	8.90 ± 1.74	8.52 ± 1.76	10.2 ± 1.89			
Δ Water content at the face, μS	Control		22.1 ± 28.6	36.9 ± 33.7	58.5 ± 39.9	0.809	0.000	0.980
	HK L-137		24.2 ± 30.9	38.3 ± 32.7	58.4 ± 43.5			
Δ Water content at the forearm, μS	Control		2.78 ± 9.03	5.58 ± 14.2	10.8 ± 16.6	0.096	0.126	0.893
	HK L-137		7.87 ± 21.5	11.4 ± 28.2	13.6 ± 26.5			
Δ TEWL at the face, g/h m^2^	Control		−0.80 ± 3.59	−0.86 ± 3.20	0.14 ± 3.65	0.050	0.164	0.934
	HK L-137		−1.69 ± 4.12	−2.19 ± 3.91	−0.81 ± 5.34			
Δ TEWL at the forearm, g/h m^2^	Control		−0.69 ± 1.64	−1.13 ± 1.65	0.29 ± 1.55	0.645	0.000	0.784
	HK L-137		−0.91 ± 1.33	−1.29 ± 1.28	0.39 ± 1.77			

ANOVA, analysis of variance; HK L-137, heat-killed *Lactiplantibacillus plantarum* L-137; TEWL, transepidermal water loss.

Values are the mean ± SD.

* P < 0.05,

† P < 0.10: significant difference of the mean value in the test group vs the control group (unpaired Student's t-test).

a Significant differences were evaluated by 2-way ANOVA (mean values, repeated measure ANOVA; changes from baseline, 2-way ANOVA).

In the published article, there was an error in Table 2 as published. The value of ΔTEWL at the forearm, g/h m^2^ in the HK L-137 group at 4 weeks was incorrectly listed as “−2.91 ±1.33.” The correct value is “−0.91 ±1.33.” The corrected Table 2 and its caption appear below.

The authors apologize for this error and state that this does not change the scientific conclusions of the article in any way. The original article has been updated.

## Publisher's note

All claims expressed in this article are solely those of the authors and do not necessarily represent those of their affiliated organizations, or those of the publisher, the editors and the reviewers. Any product that may be evaluated in this article, or claim that may be made by its manufacturer, is not guaranteed or endorsed by the publisher.

